# Predictors of Compliance to Gluten-Free Diet in Children with Celiac Disease

**DOI:** 10.1155/2014/248402

**Published:** 2014-08-28

**Authors:** Anu Garg, Rajkumar Gupta

**Affiliations:** ^1^Department of Pediatrics, Sir Padampat Mother and Child Health Institute (J.K. Loan Hospital), SMS Medical College, Jaipur, Rajasthan 302004, India; ^2^Department of Pediatrics, Subharti Institute of Medical Sciences, Meerut, Uttar Pradesh 250004, India

## Abstract

*Aim.* To identify the predictors of compliance to gluten free diet in children with celiac disease. *Methods.* 134 children in the study group were assessed for dietary compliance followed by a questionnaire based interview. Psychosocial parameters were assessed by standard Pediatric Symptom Checklist (PSC). Dietary compliant and noncompliant groups were compared and assessed for factors affecting the dietary compliance. Predictability of all of these factors was assessed using binary logistic regression analysis with backward elimination to find out the best predictors of compliance. *Results.* In the study group, 88 (65.67%) were found to be strictly compliant. Factors that were found to be significantly associated with compliance were age at presentation, nuclear families, mother's education, and parents having better knowledge of celiac disease. Parents' and child's attitude towards his having to follow a restrictive diet and child's feelings were also shown to be significantly associated with compliance. Binary logistic regression analysis with backward elimination demonstrated that age at presentation, family type, child's attitude, and child's behaviour made a significant contribution to prediction. *Conclusions.* These results will contribute to the current body of research by providing health care practitioners with a framework for better dietary instruction to ensure maximum adherence to GFD.

## 1. Introduction

Celiac disease (CD) is an immune-mediated systemic disorder elicited by gluten and related prolamins in genetically susceptible individuals and characterised by the presence of a variable combination of gluten-dependent clinical manifestations, CD-specific antibodies, HLA-DQ2 or HLA-DQ8 haplotypes, and enteropathy [1]. Recently, the prevalence of celiac disease across the European countries was shown to be 1.5% based on people who had positive biopsy and tTG results [2]. In the United States, the overall prevalence of celiac disease in children up to 5 years of age is 1 in 104 [3]. This disease is quite prevalent in India also with rates of 1 in 96 in north India [4].

Lifelong adherence to a gluten-free diet (GFD) is the cornerstone treatment of celiac disease [5]. A gluten-free diet entails strict avoidance of all products containing the proteins from wheat, barley, and rye [6]. It is strongly recommended that gluten elimination from diet must be strict and lifelong not only to control symptoms but also to improve quality of life and decrease the risk of complications [7]. Although a well-planned gluten-free diet may provide adequate nutrition, it may be restrictive. Strict adherence to gluten-free diet may be more challenging in children and adolescents than in adults. Compliance to GFD varies from 45% to 81% in children as reported by the North American Society of Pediatric Gastroenterology, Hepatology, and Nutrition [8].

Noncompliance is a major problem and the greatest challenge which the physicians face is in predicting the compliance to the gluten-free diet in children. Noncompliance may occur due to factors like temptation and not liking the taste of gluten-free food and alternative food grains [9]. In adolescents, peer pressure, unclear labelling on ready-to-eat food, and nonavailability of gluten-free food at party, marriages, and so forth have contributed to noncompliance [10]. An increasingly hectic lifestyle of teenagers has contributed to a greater reliance on packaged foods which often contain gluten, and thus making it inconvenient for them to adhere to restrictive diet [10]. Since parents are usually responsible for food preparation for children, low level of knowledge about the diet in the parents, nonavailability of gluten-free foods, and unclear labelling lead to noncompliance in children [11]. Many children experience psychological reactions to being placed on a restrictive diet (e.g., feeling deprived, depressed, angry, and anxious) which have been found to further decrease compliance [12].

This study evaluates the impact of celiac disease and the gluten-free diet on the lifestyle and well-being of children with celiac disease and their families, with the aim to identify factors affecting compliance to GFD and predictors of compliance to GFD in children with celiac disease.

This study is significant and will contribute to the current body of research by providing health care practitioners with information as to what predicts the compliance to GFD, which may be used to better understand education techniques for dietary instruction so that the children living with celiac disease have less of morbidity and achieve their normal growth potential. Participants will contribute to the understanding of celiac disease and the challenges individuals face with the gluten-free diet.

## 2. Material and Methods

This study was conducted in the tertiary care hospital of the Department of Pediatrics, SMS Medical College, Jaipur, Rajasthan, India. During a period of 1 year starting from October 1, 2011, till September 30, 2012, 150 consecutive celiac disease children visiting the gastroenterology super-specialty clinic were studied. These children visited the clinic for growth monitoring and compliance assessment. 134 consecutive children out of these meeting the following inclusion criteria were enrolled in the study:

1. patients aged between 2 years and 15 years,
2. children diagnosed with celiac disease as per Revised ESPGHAN criteria for diagnosis of celiac disease 1990 [13],
3. those on gluten-free diet for more than 6 months.

Exclusion criteria applied were (1) any child less than 2 years and more than 15 years of age, (2) those who did not have a documented positive serology and/or biopsy suggestive of celiac disease as per revised ESPGAN criteria 1990, (3) those on gluten-free diet for less than 6 months, and (4) those children whose parents did not consent to be included in the study.

All children enrolled in the study after signing of the written informed consent form were evaluated for dietary compliance based on a 5-day dietary recall form. A child who had taken even one food article containing gluten in last 5 days was considered noncompliant and those who had strictly taken no gluten in their diet in that period were considered compliant. Diet recall was done by parents for children in preschool age up to 5 years since parents were the only one giving the eatables to these children. Children, above 5 years of age, going to school and interacting with peers, were actively involved in the dietary recall along with the parents.

After the dietary assessment, children and their parents were subjected to an interview by the investigator who was blinded for the compliance status of these children. Interview consisted of a self-administered questionnaire which had questions related to demographic profile, history of illness, parents' knowledge and understanding of disease, barriers to compliance and effect of celiac disease on feelings of children, eating out, and travel. The questionnaire was developed from 4 previously published studies [11, 14–16] and it was pretested and validated in 20 children in the past in the paediatric gastroenterology unit of Kalawati Saran Children's Hospital, Delhi, India [9].

To assess psychosocial problem, standard Paediatric Symptom Checklist containing 35 items was added to the questionnaire [14]. The Pediatric Symptom Checklist (PSC) is used to screen psychosocial problems in a child, by using a parent completed screening questionnaire as a part of routine primary care visit [14]. It is a screening tool that reflects the parent's impression of his or her child's psychosocial functioning [17, 18]. Each PSC item is rated as

1. never—0,
2. sometimes—1,
3. often—2.

Item scores are summed and the total score is recorded into a dichotomous variable. PSC score of 28 or higher for children aged 6 years and above, and 24 or higher in children between 2 and 6 yr is taken as emotional and psychosocial impairment. The items for which no answer is given were scored 0. If 4 or more items are unanswered, questionnaire was treated as invalid.

During the final data analysis, based on the assessment of compliance to gluten free diet, the group was subdivided into dietary compliant group and dietary noncompliant group. The factors that were found associated with compliance significantly were found out. This collected data was analysed in Microsoft excel 07 and Primer 5.00 and classified as per aims and objectives. The data was found not normal hence nonparametric methods were used. Inference was drawn using chi-square test and Mann-Whitney *U* test to compare the compliant and noncompliant groups. *P* value of <0.05 was considered significant.

Binary logistic regression analysis with backward elimination was applied using software PASW18 (trial version), to assess predictability of all the variables used in the study. Dietary compliance was used as dependant variable and various factors studied for association with compliance were used as factors affecting the dependent variable.

## 3. Results

In the study group of 134 children bearing the characteristics as shown in Table 1, 88 children (65.67%) were compliant and 46 (34.33%) were noncompliant. Compliance was higher in children up to 9 years of age as compared to that in children aged above 9 years. Females were slightly more compliant than males; however the difference was not statistically significant (*P* = 0.528). Compliance was significantly associated with higher level of mother's education (*P* = 0.001) and type of family, whether nuclear or joint, with children living in nuclear families being more compliant (*P* = 0.05). As evident from Table 1, 93.75% of children with graduate and postgraduate mothers were compliant. Only 78.05% of children with mothers educated up to the 10th class or nongraduate were compliant and only 53.25% of children with mothers educated up to the 8th class were compliant. Among children from nuclear families, 73.61% were compliant as compared to only 56.45% children from joint families who were compliant to gluten-free diet.

Number of children in compliant and noncompliant group was not significantly different in relation to age at diagnosis of celiac disease (*P* = 0.143), sex of the child (*P* = 0.528), per capita family income (*P* = 0.215), and number of siblings (*P* = 0.983). Father's education also was shown not to affect compliance significantly (*P* = 0.248). Despite being an important factor, the symptom with which the child came when he was diagnosed as having celiac disease, whether gastrointestinal or nongastrointestinal, did not correlate with compliance significantly (*P* = 0.951) in our study.

Considering that since parents are the ones responsible for feeding the child, their knowledge and general awareness about the disease do significantly relate to compliance. As shown in Table 2, parents of children in the compliant group had better understanding of the nature of the disease and were also very evidently more aware about the treatment and better identified the gluten-free food articles. There was a statistically significant difference among the parents of compliant and noncompliant children with respect to their knowledge about celiac disease.

As seen in Table 3, comparing parents' attitude towards their child's disease, there was significant difference between compliant and noncompliant groups with *P* ≤ 0.001. More than half of the parents of the noncompliant children perceived that GFD was a burden on their budget while this number was only 19.32% in the compliant group. Dietary noncompliance was significantly more in children whose parents felt that preparing a special diet everyday was a (psychological) burden on them (54.35% in noncompliant group versus 20.45% in compliant group) and in those who were hesitant to discuss the child's condition with others and were not interacting with other parents of celiac disease in the gastroenterology clinics or awareness camps held in the hospital.

**Figure 1:**
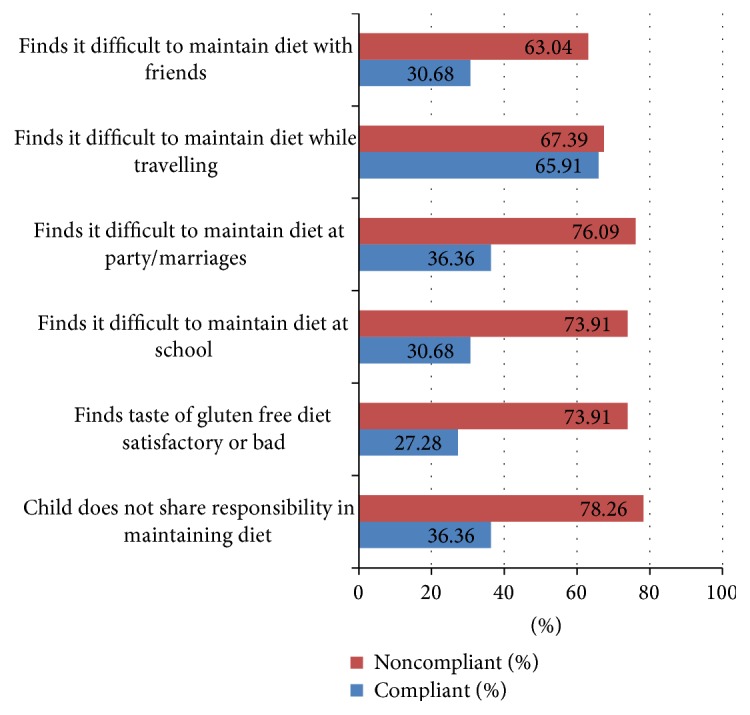
Child's attitude towards GFD and its association with compliance.

As shown in Figure 1 and Table 4, child's attitude towards his restrictive life style is shown to be as much related with dietary compliance as that of parents'. 56.82% of children in compliant group found keeping to GFD easy, while only 19.57% of noncompliant children found it easy to keep to a gluten-free diet. 63.64% children in the compliant group shared the responsibility with their parents in maintaining gluten-free diet as compared to only 21.74% children in the noncompliant group who did so. As seen in Table 4, a subjective feeling of finding the taste of GFD very good and good was seen in 72.72% of compliant children while it was seen in only 25.92% of noncompliant children. It is evident that a child's positive attitude towards his restrictive diet goes in sync with his adherence to GFD. Although both groups reported difficulties to maintain compliance at family party/marriage, at school, however this feeling of difficulty to adhere to GFD was significantly less in compliant group as shown in Table 4. Travelling has come out as an issue in our study where all the children face difficulty to maintain GFD. This points towards a scarcity of packaged ready to eat properly labelled gluten-free eatables' availability in market.

Table 5 shows that the feeling of “anger to follow a special diet,” feeling of “embarrassment to bring gluten-free food to parties,” and feeling of being “left out of activities at school because of disease” are significantly higher in noncompliant children than compliant ones. 7.95% of compliant children felt left out of activities at school or friend's place all or most of the time while this feeling is seen in 47.8% of noncompliant children all or most of the time. 19.32% compliant children felt angry all or most of the time on having to follow a special diet. 69.57% children in noncompliant group felt so all or most of the time. Our study has shown that how a child feels about his situation is associated with compliance significantly (*P* ≤ 0.001).

Comparing the PSC scores in both groups, mean PSC score for compliant group was lower, that is, 8.3 as compared to 14.6 in noncompliant group. In both compliant and noncompliant groups, 1 patient had significant PSC score indicating screen test positive for social, emotional, and psychological impairment, thus showing that chances of psychosocial impairment exist in both compliant and noncompliant children, being more in noncompliant ones.

**Figure 2:**
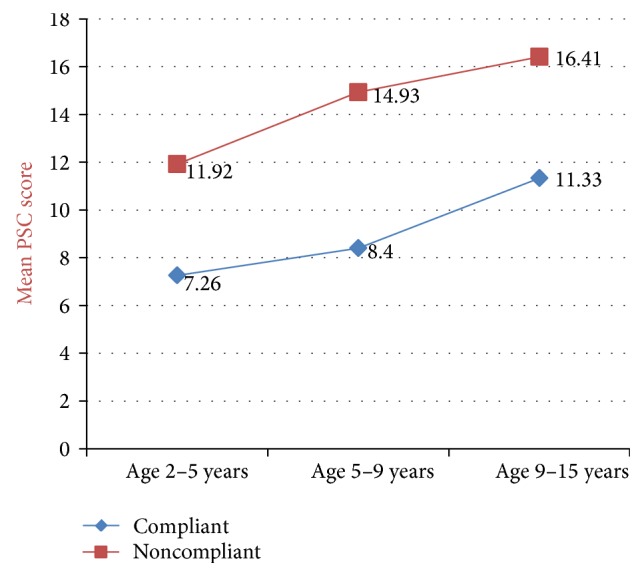
Mean PSC scores in various age groups.

In Figure 2, the mean PSC score was seen to be increasing with age in the children suffering from celiac disease; hence an older child is more at risk of psychosocial impairment. And this increase in PSC scores with age is seen both in compliant and noncompliant group emphasizing that all children whether compliant or not are at risk for psychosocial impairment. Maximum score was seen in children >9 years of age.

A binary multivariable logistic regression analysis with backward elimination was conducted to narrow down predictors of dietary compliance using all the factors assessed previously (whether found to be significantly associated or not), namely, age, sex, age at presentation, presenting symptom, that is, GI or non-GI, father's education, mother's education, family type, per capita income, number of siblings, parental knowledge, parental attitude, child's attitude, child's behaviour and PSC score as predictors, and using “compliance” as dependent variable.

The Wald criterion demonstrated that only age at presentation (with *P* value = 0.004), family type (with *P* value = 0.0097), child's attitude (with *P* value = 0.0003), and child's behaviour (with *P* value = 0.0195) made a significant contribution to prediction. Other factors associated with compliance to GFD were not significant predictors (Table 6).

Seeing the odds ratio of the significant predictors, it was demonstrated that the best predictor of compliance is child's attitude which, if favourable, makes the child 7 times more likely to be compliant. Second best predictor is child's behaviour, and third is family type. Children with a positive outlook and feelings about their condition are 4 times more likely to be compliant; similarly children of nuclear families are 4 times more likely to be compliant. Odds ratio for age is 0.75, that is, with each unit increase in age (one year) chance of compliance becomes 25% less likely.

## 4. Discussion

Complying with GFD can be extremely challenging for children with celiac disease. 65.67% of the children in our study were found to be dietary compliant. This was in harmony with the rates of compliance as seen in various other studies done in India and outside. Strict dietary compliance was shown to vary from 45% to 81% in children by Hill et al. [8], 95% in a Canadian study on children <16 years of age by Rashid et al. [15], and 75% by Chauhan et al. in a study done on children 2–17 years of age, in north India in 2010 [9].

Whereas young children with celiac disease may adhere to a gluten-free diet because of parental influence, the situation remains complex in adolescents. Our study found decreased dietary compliance above 9 years of age. The percentage of compliant children dropped from 75.92% in children >2–5 years to 41.37% in children above 9 years of age. These results are in accordance with Ljungman and Myrdal [16], who also reported compliance rates of 93% at 12 years of age decreasing to 76% in 15–17 yr age group. Various reasons which may be responsible for increasing noncompliance with increasing age include increased social interaction, increasing peer group pressure, increased outdoor activities, and need for experimentation. Compliance was not significantly associated with the sex of the child in our study, just as in a study by Errichiello et al. [10].

This low global level of adherence to a GFD in children with celiac disease is troubling given the known morbidity and mortality associated with long-term untreated celiac disease and the lack of any other effective treatment. Effective counselling about the diet is the single most important factor to ensure the required restriction in diet among these patients. Our study enumerates factors which are significantly associated with compliance to GFD and understanding the predictors of compliance will help the clinician to target the problem areas and ensure maximum compliance among children.

Mother's education is found as a significant factor related with the compliance in our study. It may be because mother is responsible for buying and preparation of food items. With her knowledge, she is able to identify better which food stuff is gluten free. Anson et al. [11] (1990) also found that maternal education is important factor associated with compliance.

A statistically significant association of compliance was seen with nuclear family which is coherent with results of Chauhan JC et al. in 2010 [9]. Joint family may lead to noncompliance as with many people around the child having all varieties of food, tempts the child and leads him to consume gluten containing food.

Our study also highlights that higher degree of compliance is noted when parents have better knowledge about celiac disease and the gluten containing items, understand importance of gluten-free diet for their child's overall growth and development, and are able to distinguish gluten containing from gluten-free food so that they handle the menu better. Anson et al. [11] also showed similar correlation of parental knowledge and dietary compliance. There is more evidence that compliance with the gluten-free diet is improved in those who are more knowledgeable about celiac disease and the diet [19]. This study also shows that a parents' positive attitude towards the child's condition is associated with higher compliance. Dietary noncompliance is more common when preparing gluten-free food items is considered both a burden on self and financial burden by parents. Similar finding also has been reported from Anson et al. [11]. So counselling aiming to increase the knowledge about the disease and awareness of parents regarding cheap and acceptable alternatives to wheat and easy to cook gluten-free food recipes will help ensure compliance to GFD in children.

Child's positive attitude towards his condition has also come out as a significant factor associated with higher degree of compliance. Difficulty in maintaining dietary compliance at school and at family party and marriages was higher in dietary noncompliant group compared to the dietary compliant. Gluten containing food as main dietary item served at above places was a problem for children in both dietary compliant and noncompliant groups. These results highlight that the need of widespread availability of gluten-free food and more clear and apparent labelling of gluten-free items are of as much importance as proper counselling and reinforcement during subsequent visits.

The study results show that dietary restriction has effect on child's feelings and social activities like eating out and travelling. Rashid et al. in their study in Canada also reported that more than 50% of children felt left out of activities at school and had problems related to compliance [15]. Anson et al. have reported similar barriers to compliance in relation to child's feelings [11]. These results indicate that negative feelings in some children owing to their dietary restriction are associated with noncompliance in these children. This is the area where the counselling of the treating doctor is utmost important so that a child can be made to better accept his situation and the efforts of the parents of the child can be better channelized to achieve this.

Out of all these various factors studied for association to adherence to GFD, our study narrowed down four factors which can serve most closely as the predictors of compliance. Child's attitude (best predictor with OR = 7, *P* value = 0.0003), child's behaviour (with OR = 4, *P* value = 0.0195), family type (with OR = 4, *P* value = 0.0097), and age at presentation (with OR = 0.75, *P* value = 0.0004) made a significant contribution to prediction of compliance.

One child in both compliant and noncompliant group in our study has screened positive for psychosocial problems and so such problems can be encountered in both compliant and noncompliant children. Addolorato et al. have shown that anxiety is present in celiac disease subjects as a reactive form which decreases with gluten-free diet; however depressive symptoms still persist in patients on GFD [20]. Ciacci et al. in their study have also reported that anger is the predominant emotion which induced patients to transgress [21].

PSC scores are seen to increase as the age increases, more so in noncompliant children. Maximum score was seen in children >9 years of age which is fairly understandable as this is the age when children interact with people other than their parents and develop a defiant attitude, give in to peer pressure, and develop a need for experimentation. The need for psychological support to all patients when they are put on gluten-free diet and continued psychological support through a professional especially to those above 9 years of age is thus emphasised here.

Our study adds to the available data regarding the significant factors that play a role in compliance to GFD. However, a few limitations of this study are to be mentioned. First, compliance was not confirmed with concurrent histological and/or serological evaluation. However it is not clear if biopsy provides a better assessment of long-term compliance than nutritional evaluation. In fact, prior studies showed only a modest correlation of histology with clinical presentation or assessed dietary adherence [22, 23]. While histological and serological relapse has understandably been used to assess compliance, in some studies a more practical and noninvasive approach has been used where compliance was assessed subjectively on the basis of dietary recall by parents and children in last few days. In a study by Mustalahti in 2002, the patients' self-concept of long-term adherence to the gluten-free diet was evaluated by using a visual analogue scale. Patients indicated their level of adherence by marking a scale anchored at 0% and 100% compliant. At the end of the study, all patients were again asked to complete a 4-day food record [24]. Jadrešin et al. 2008 in their study defined compliance with gluten-free diet as strict, semistrict, and not on gluten-free diet based on 7-day recall of patient's intake of amount of gluten assessed subjectively by patient himself or guardian [25]. Spatola in his study in 2014 has also used a 4-day dietary record to assess compliance wherein those consuming no gluten-containing item in these 4 days were considered compliant [26]. Therefore we have used a noninvasive method of dietary recall of 5 days to assess compliance. Due to lack of reliable tools it is challenging to uncover minor dietary lapses or inadvertent gluten intake. To ensure compliance status of our study group, all subjects had been interviewed repeatedly in their follow-up visits in our Gastroenterology super-speciality clinic by trained dietician and senior consultants, to uncover hidden transgressions and establish compliance status based on clinical interview besides the 5-day food record.

Also, results from a cross-sectional study cannot establish causality. Bidirectionality of evidence is a major limitation in the current study design. However, the results of this study point to a number of areas, both obvious and obscure, that may be productive targets for interventions aimed at improving dietary adherence in individuals and our future studies will aim to establish the validity of these associations.

## 5. Conclusion

Celiac disease is a common problem in most parts of world. This is because most doctors currently suspect and diagnose the disease in children presenting with nongastrointestinal symptoms as well as atypical presenters and in at risk asymptomatic children by active case-finding strategy (serologic testing for celiac disease in patients with symptoms or conditions closely associated with celiac disease) [27].

For many years, physicians have focused on the diagnosis and molecular and cellular markers of celiac disease, with scarce attention being given to the care and well-being of the patient. Patients with celiac disease should be monitored regularly for residual or new symptoms, adherence to gluten-free diet, and assessment for complications. Normal growth and development are achievable on a gluten-free diet and should be goals for monitoring children with celiac disease [28]. As per ACG, monitoring of adherence to gluten-free diet should be based on a combination of history and serology [6].

Hence for monitoring of compliance to the diet by history, it may be stated that interventions specially aiming at improving the child's understanding and acceptability of celiac disease to promote a favourable attitude and positive feelings may increase the compliance to gluten-free diet. Children should receive support through school health teams and health facilities and support groups. It may be stated that easy availability of palatable, cheap, and socially acceptable gluten-free food and better labelling of food may increase compliance to gluten-free diet. It is evident from our results that if the child lives in a joint family, he needs more of follow-up visits and more efforts from physician's side to ensure compliance as he is more likely to defy the diet. Also since age at presentation predicts compliance, the responsibility lies on the medical fraternity to identify the typically and atypically presenting children and put the child on gluten-free diet at an age which is less affected by the peers and such that the child inculcates the dietary habits required of him, for life. There is a lot of scope for establishment of celiac support groups in India wherein the doctors, NGOs, and volunteering individuals can work together to provide the necessary psychological support to the children, where parents are informed of the varied recipe options and upcoming treatments, and a routine assessment can be done from time to time for any psychosocial impairment to all the children, especially those above 9 years of age.

## Figures and Tables

**Table 1:** Demographic characteristics of the study group.

Parameters	Compliant group	Noncompliant group	*P* value
Number	88 (65.67%)	46 (34.33%)	
Median age (yrs)	6 yrs. (range 2.5–14.5 yrs.)	8 yrs. (range 2.5–15 yrs.)	0.002
Sex			
Male	51 (62.96%)	30 (37.04%)	0.528
Female	37 (69.8%)	16 (30.2%)	
Age group			
a >2–5 yrs	41 (75.92%)	13 (24.08%)	
b >5–9 yrs	35 (68.62%)	16 (31.38%)	0.006
c >9–15 yrs	12 (41.37%)	17 (58.63%)	
Father's education			
Up to 8th	25 (56.82%)	19 (43.18%)	0.248
10 to nongraduate	32 (66.67%)	16 (33.33%)	
Graduate and post graduate	31 (73.81%)	11 (26.19%)	
Mother's education			
Up to 8th	41 (53.25%)	36 (46.75%)	0.001
10 to nongraduate	32 (78.05%)	9 (21.95%)	
Graduate and post graduate	15 (93.75%)	1 (6.25%)	
Type of Family			
Joint	35 (56.45%)	27 (43.54%)	0.05
Nuclear	53 (73.61%)	19 (26.38%)	
Per Capita income per month			
Up to 2000 Rs	51 (60.71%)	31 (39.29%)	0.215
2000-up to 5000 Rs	35 (72.92%)	13 (27.08%)	
>5000 Rs	2 (100%)	0 (0%)	

**Table 2:** Parental knowledge and compliance to gluten-free diet.

Question	Response	Compliant (%)	Noncompliant (%)	*P* value
Knows correctly which part is affected	Y	34 (38.64%)	4 (8.70%)	<0.001
	N	54 (61.36%)	42 (91.3%)	

Knows mainstay treatment	Y	77 (87.50%)	29 (63.04%)	0.002
	N	11 (12.50%)	17 (36.96%)	

Believes occasional transgression is harmful	Y	69 (78.41%)	14 (30.43%)	<0.001
	N	19 (21.59%)	32 (69.57%)	

Knows lifelong restriction	Y	40 (45.45%)	9 (19.37%)	0.006
	N	48 (54.55%)	37 (80.43%)	

Identifies gluten-free items	a = identifies all 5 items	50 (56.82%)	11 (23.91%)	<0.001
	b = identifies 3-4 items	31 (35.23%)	18 (39.13%)	
	c = identifies <3 items	7 (7.95%)	17 (36.96%)	
	d = chooses gluten containing food as gluten free	0 (0.0%)	0 (0.0%)	

**Table 3:** Compliance in relation to parents' attitude.

Question	Response	Compliant (%)	Noncompliant (%)	*P* value
Finds burden on budget	Heavily	17 (19.32)	22 (47.83)	<0.001
	Fairly	43 (48.86)	21 (45.65)	
	Hardly	28 (31.82)	3 (6.52)	

Feels burden on self	Y	18 (20.45)	25 (54.35)	<0.001
	N	70 (79.55)	21 (45.65)	

Cooks food once or more than once	>Once	81 (92.05)	26 (56.52)	<0.001
	Once	7 (7.95)	20 (43.48)	

In contact with other parents of children with celiac disease	Y	30 (34.09)	6 (13.04)	0.016
	N	58 (65.91)	40 (86.96)	

**Table 4:** Compliance in relation to child's attitude.

Question	Response	Compliant (%)	Noncompliant (%)	*P* value
Finds keeping diet difficult	Difficult	2 (2.27)	12 (26.09)	<0.001
	Fairly difficult	36 (40.91)	25 (54.35)	
	Easy	50 (56.82)	9 (19.57)	

Child shares responsibility	Y	56 (63.64)	10 (21.74)	<0.001
	N	32 (36.36)	36 (78.26)	

Finds taste of gluten-free diet	Bad	1 (1.14)	11 (23.91)	<0.001
	Satisfactory	23 (26.14)	23 (50)	
	Good	62 (70.45)	11 (23.91)	
	Very good	2 (2.27)	1 (2.1)	

Finds it difficult to maintain diet at school	Y	27 (30.68)	34 (73.91)	<0.001
	N	61 (69.32)	12 (26.09)	

Finds it difficult to maintain diet at party/marriage	Y	32 (36.36)	35 (76.09)	<0.001
	N	56 (63.64)	11 (23.91)	

Finds it difficult to maintain diet while travelling	Y	58 (65.91)	31 (67.39)	0.984
	N	30 (34.09)	15 (32.61)	

Finds it difficult to maintain diet with friends	Y	27 (30.68)	29 (63.04)	<0.001
	N	61 (69.32)	17 (36.96)	

**Table 5:** Child's behaviour as a barrier to compliance.

Parameter	Compliant Group (%)					Noncompliant Group (%)					*P* value
	A	B	C	D	E	A	B	C	D	E	
Felt left out of activities at school or friends home	0 (0)	7 (7.95)	38 (43.18)	42 (47.73)	1 (1.14)	1 (2.17)	21 (45.63)	15 (32.61)	9 (19.57)	0 (0.0)	<0.001

Felt different from other kids	0 (0)	3 (3.41)	35 (39.77)	50 (56.82)	0 (0.0)	5 (10.87)	19 (41.3)	13 (28.26)	9 (19.57)	0 (0.0)	<0.001

Felt embarrassed to bring gluten-free foods to parties	1 (1.14)	8 (9.09)	15 (17.05)	64 (72.73)	0 (0.0)	5 (10.37)	18 (39.13)	7 (15.22)	12 (26.09)	4 (8.7)	<0.001

Felt angry about following a special diet	0 (0.0)	17 (19.32)	42 (47.73)	29 (32.95)	0 (0.0)	20 (43.48)	12 (26.09)	11 (23.91)	3 (6.52)	0 (0.00)	<0.001

Felt they were not invited out	1 (1.14)	8 (9.09)	20 (22.73)	52 (59.09)	7 (7.95)	8 (17.39)	2 (4.35)	5 (10.87)	25 (54.35)	6 (13.04)	0.003

A. All the time.

B. Most of the time.

C. Some of the time.

D. Never.

E. Not answered.

**Table 6:** Predictors of compliance.

	Wald test	Odds ratio	*P* value
Age	12.3784	0.7257	0.0004
Family type	6.6856	4.3807	0.0097
Child's attitude	13.1990	7.0192	0.0003
Child's behaviour	5.4578	4.7383	0.0195
